# Predilection of Segmental Glomerulosclerosis Lesions for the Glomerulotubular Junction Area in Type 1 Diabetic Patients: A Novel Mapping Method

**DOI:** 10.1371/journal.pone.0069253

**Published:** 2013-07-25

**Authors:** Behzad Najafian, Michael Mauer

**Affiliations:** 1 Department of Pathology, University of Washington, Seattle, Washington, United States of America; 2 Departments of Pediatrics University of Minnesota, Minneapolis, Minnesota, United States of America; 3 Department of Medicine, University of Minnesota, Minneapolis, Minnesota, United States of America; Hirosaki University Graduate School of Medicine, Japan

## Abstract

The location of segmental glomerular lesions in relation to the vascular or tubular pole may have diagnostic or prognostic significance. We have developed a model-based method to estimate the distance from a glomerular lesion to a given landmark (vascular or tubular pole) or the glomerular center and applied this to biopsies from 5 microalbuminuric, 5 normoalbuminuric and 7 proteinuric type 1 diabetic patients and 5 normal controls. The distance from each glomerular adhesion to the glomerulotubular junction was measured and divided by the glomerular radius, allowing comparability among different glomeruli, assuming a spherical shape for Bowman's capsule, an assumption which was validated. The frequency of adhesions in 6 glomerular zones with equal height (zone I adjacent to the glomerulotubular junction and zones II–VI progressively farther away) was determined: 59% of adhesions were in zone I, 15% in zone II, 16% in zone III, 7% in zone IV and 3% in zone VI (adjacent to the hilus). In glomeruli with only one adhesion, 82% of these were in zone I. This new method accurately localizes segmental lesions within glomeruli and revealed a marked predilection in type 1 diabetic patients for segmental sclerosis to develop at the glomerulotubular junction.

## Introduction

The position of segmental glomerular lesions may be of diagnostic or prognostic significance. For example, segmental sclerosis at the glomerulotubular junction (GTJ) in idiopathic focal segmental glomerulosclerosis (FSGS), the so called tip lesion, has been associated with a better prognosis [1]–[3]. Localization of segmental glomerular lesions usually requires serial sectioning or three-dimensional (3-D) reconstruction, unless the lesion happens to be present on the same section as an anatomic landmark (GTJ or vascular pole). We developed a model-based method for mapping glomerular lesions and estimating their distances from a given landmark or the glomerular center. We previously showed that tip lesions are frequent in proteinuric type 1 diabetic (T1DM) patients [4], [5]. In order to see if there is a predilection for tuft adhesions to be localized in the GTJ region, this method was applied to biopsies studied in our previous publication [4]. A practical example is provided to illustrate usage of the grid and equations.

## Results

Renal biopsies from 5 normoalbuminuric, 5 microalbuminuric and 7 proteinuric T1DM patients and 5 normal controls were studied. The demographic and clinical characteristics of the subjects are summarized in Table 1. There were 10 to 13 (11.0±1.2) complete glomeruli (with intact Bowman's capsule) per biopsy on serial sections. None of the 50 glomeruli from the control subjects contained adhesion or segmental sclerosis. One of 50 (2%) glomeruli in normoalbuminuric, 2 of 50 (4%) glomeruli in microalbuminuric and 64 of 77 (83%) glomeruli in proteinuric T1DM patients had adhesions and segmental sclerosis. Where present, there were 1.5±1.0 (range 1 to 6) adhesions per glomerulus, with most of these glomeruli (65%) having only one adhesion.

**Table 1:** Demographic and clinical characteristics of cases.

Group	F/M	Age	DD	HTN (Y/N)	AER	GFR
**C**	2/3	34 [25–40]	-	0/5	-	-
**NA**	3/2	30 [22–36]	12 [10–15]	0/5	8 [2–10]	111±5
**MA**	4/1	35 [17–49]	17 [10–33]	1/4	42 [25–118]	92±18
**P**	3/4	37 [26–49]	25 [16–30]	7/7	387 [226–3263]	66±27

Abbreviations: C = non-diabetic control; NA = normoalbuminuric; MA = microalbuminuric; P = proteinuric type 1 diabetic patients; F = female; M = male; DD = diabetes duration; HTN (Y/N) = hypertension (yes/no); AER = albumin excretion rate (µg/min); GFR = glomerular filtration rate (ml/min/1.73 m^2^). Age, DD and AER values are median [range], and GFR values are mean ± SD.

**Figure 1:**
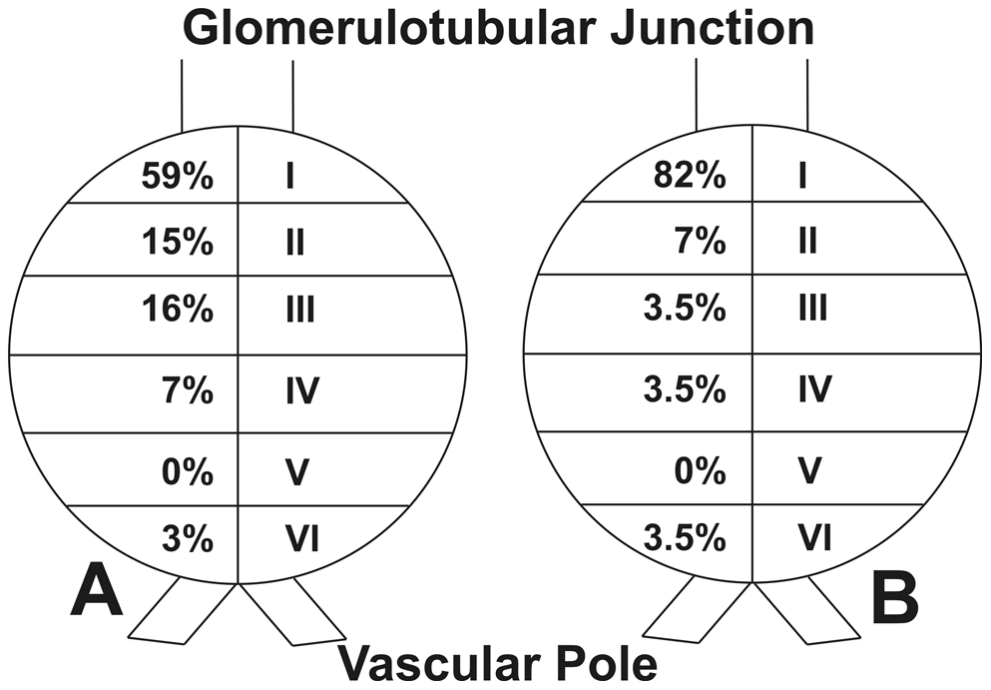
Distribution of adhesions of segmental sclerosis and adhesions of tuft to Bowman's capsule in relation to the glomerulotubular junction and the vascular pole. (**A**) in all glomeruli and (**B**) in glomeruli with only one segmental sclerotic lesion.

In order to estimate the distance of adhesions from GTJ, a model-based method was used. In order to illustrate distribution of adhesions to the Bowman's capsule, the distances from the GTJ were expressed relative to the glomerular radius and each adhesion was mapped on a virtual spherical Bowman's capsule divided into 6 zones with zone I adjacent to GTJ and zone VI on the opposite side (see methods and **Figure 1**). The volume contained within the Bowman's capsule estimated using the Cavalieri principle [6] (3.4±1.2×10^6^ µm^3^) or calculated from the sphere radius obtained on serial sections (3.8±1.8×10^6^ µm^3^) were strongly correlated (r = 0.82; p = 0.0001) and not statistically different, this validating the assumption of spherical shape for Bowman's capsule in these glomeruli.

Over half of adhesions were located adjacent to the GTJ (zone I, **Figure 1**). Since any area of Bowman's capsule occupied by an adhesion would not be available for additional adhesions, mapping was also performed separately for glomeruli containing only one adhesion. Eighty-two percent of these adhesions were located in zone I (**Figure 1**). The relative distance of adhesions to GTJ was 0.63±0.48 glomerular radial length. There was no statistically significant relationship between distances of adhesions to GTJ and AER or GFR.

## Discussion

Previous studies of the position of segmental glomerular lesions are mostly descriptive and based on observations of single or serial sections [7]–[10]. Newbold and Howie developed a method to graphically illustrate the position of segmental lesions that were present in sections which also included the vascular pole [9]. They showed that most segmental lesions in biopsies with the tip lesion variant of FSGS, were in the GTJ region, while most lesions of glomeruli from patients with a single kidney were either around the GTJ or the vascular pole, and the distribution of vasculitic type lesions was random in glomeruli [9]. A similar approach suggested a random distribution of mesangial nodules in glomeruli from diabetic patients [11]. The application of the present method is not limited to sections containing both the segmental lesion and a given anatomic landmark and allows for a real three-dimensional (3-D) analysis. Moreover, this new method not only provides a graphical representation of the distribution of segmental lesions, but also provides a quantitative measure of the distance of each lesion from a landmark or from the glomerular center. Remuzzi *et al* provided a schematic representation of the distribution of segmental sclerosis lesions using serial sections of glomeruli from adriamycin treated rats [12]. Their method although accurately counted the number and estimated the volume of sclerotic lesions, did not provide information on their location. Perico *et al* adopted a similar methodology to study cyclosporine-induced glomerulosclerosis in a rat renal transplant model [13]. Remuzzi *et al*, using computer-based image processing, examined 3-D reconstructions of glomeruli with segmental sclerosis from patients that had undergone subtotal nephrectomy [14]. Although these investigators marked the vascular pole on the images of the studied glomeruli, they did not provide any information regarding the relationship or distance of the lesions from this landmark. The present method, while requiring serial sectioning of intact glomeruli within the biopsy specimen, does not require glomerular 3-D reconstruction, which often requires special softwares and expertise. However, the proposed methodology and equations can be applied equally well to 3-D reconstructed images, as well as to optical sections obtained by confocal microscopy. Moreover, this method can be used to determine the location of any lesion within a glomerulus in relation to any identifiable glomerular reference point, so long as these are of sufficient size as to have little or no possibility of being missed between the serial sections.

The present model-based method and equations were developed based on the assumption that the shape of Bowman's capsule is spherical. In order to validate this assumption, we compared volumes contained within Bowman's capsule using the Cavalieri principle, a shape-independent method [6], to volumes calculated from the radius obtained from the number of serial sections passing through the glomerulus and section thickness. These volumes were not statistically different and were highly correlated, validating the model shape assumption. Our previous studies using a two profile area method and a maximal profile area method to estimate glomerular tuft volumes, both based on a spherical shape assumption for the glomerular tuft [6], [15], correlated well with the shape-independent Cavalieri measurements. Nevertheless, Bowman's capsule generally retains its spherical shape better than the glomerular tuft, perhaps because it is supported by the surrounding tissues. .

We have previously shown that segmental glomerulosclerosis is a late finding in T1DM, almost entirely restricted to proteinuric patients who already have advanced glomerular basement membrane thickening and mesangial expansion [4]. The present study showed that these lesions have a remarkable predilection to occur close to GTJ, a feature that we believe should be recognized as one of the characteristic features of the morphologic expressions of diabetic nephropathy in T1DM patients. These special features should be considered in evaluating whether segmental sclerosis lesions in diabetic animal models do, in fact, mimic human diabetic glomerulopathy. Applying this method to other glomerular lesions (necrosis, endocapillary proliferation, etc.) and conditions could provide important pathophysiologic insights.

## Methods

### Tissue handling and digital imaging

**Figure 2:**
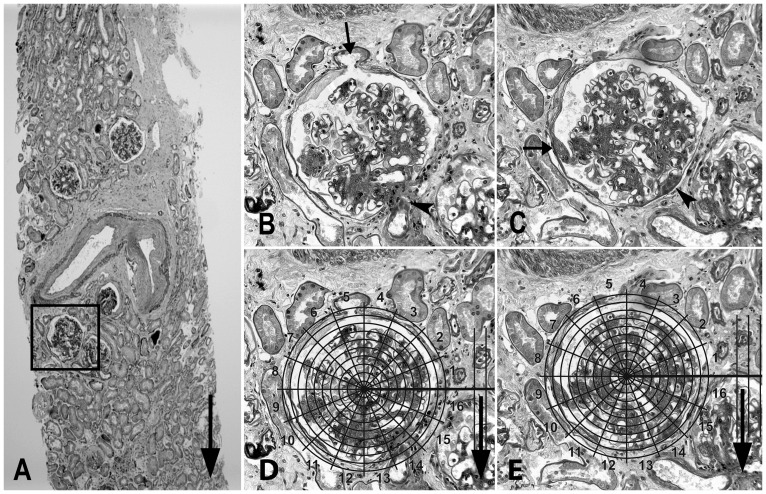
Applying the target grid in two parallel sections of known distance to map segmental lesions in glomeruli. (**A**) Low magnification (120×) image of a section, used to draw the reference axis (vertical arrow on the right side of the section) in a separate layer in Photoshop. This axis is then transferred to the high magnification (1230×) images of serial sections from a sampled glomerulus (outlined in the black square). (**B and C**) Two serial sections, 15 µm apart, from the glomerulus selected in (A). Section (**B**) contains the glomerulotubular junction (arrow) and the vascular pole (arrowhead) of the glomerulus, while section (**C**) contains the adhesion (arrow) and a small portion of the vascular pole (arrowhead). (**D and E**) A target grid (see **Figure 3**) is superimposed on the same glomerular profiles shown in (B) and (C), respectively. (**D**) The grid is centered on the Bowman's capsule center of profile (B) and its parallel guidelines are aligned with the reference axis (vertical arrow) The distance class from the target center to the glomerulotubular junction is “10”, and the angle class between the origin axis (bold horizontal line) and the glomerulotubular junction is “5”. (**E**) The grid is similarly superimposed on profile (C). The distance class of adhesion from the target center is “9”, and the angle class between the origin axis and the adhesion is “9” as well (see the practical example in **Methods**).

All research biopsies used in this study were performed with permission of the Committee on the Use of Human Subjects in Research at the University of Minnesota and after written informed consents were obtained. All research biopsies used in this study were performed with permission of the Committee on the Use of Human Subjects in Research at the University of Minnesota and after informed consents were obtained. Paraffin embedded, Zenker fixed renal biopsies were serially sectioned at 5 µm. Consistency of section thickness are regularly checked in our laboratory by serial cutting of a test paraffin block using a microtome, measuring the total advancement of the block using a sensitive microcator (Mitutoyo, DC-112 MEB, Japan) and dividing the total advancement by the number of sections obtained. Sections were sequentially mounted on slides and stained with periodic acid Schiff. Digital imaging was performed using a Spot Insight digital camera (Diagnostic Instruments Inc, USA) and Adobe Photoshop CS3 extended (version 10.0, Adobe Systems Inc) was used to view images and superimpose grids and axes using the layer function. Complete glomeruli with intact Bowman's capsules on serial sections were selected. Low magnification (120×) images were obtained and used to draw the longitudinal axis of each section (i.e. the biopsy core axis) on a separate layer in Adobe Photoshop (**Figure 2**). This axis, hereafter called “reference axis”, was used to assure correct alignment of serial sections when performing measurements (see below). Serial section images of sampled glomeruli were obtained at 1,230×. The reference axis of each glomerulus was transferred from the low magnification image.

The above magnifications are final magnifications using 4× and 40× objectives calculated based on images from a calibration slide.

### Target grid measurements

**Figure 3:**
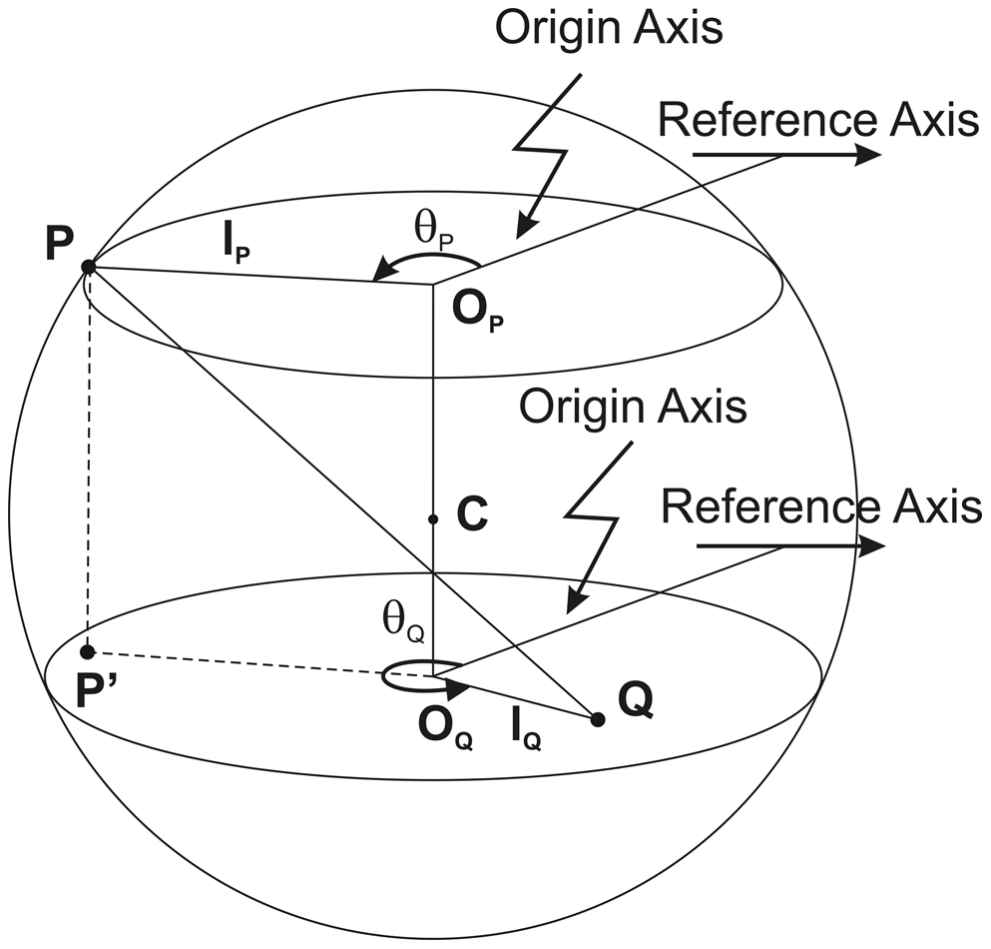
The target grid used to map segmental lesions in glomeruli. The target grid with 16 equal angular divisions (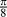 each) from the origin axis (bold line) and 10 equal distance divisions (10 µm each) from the grid center. Four parallel lines at the end of the origin axis help to orientate the origin axis perpendicular to the reference axis (arrow).

The middle sections passing through the anatomic landmark of interest (i.e, GTJ), and the lesion (i.e. adhesion of the glomerular tuft to Bowman's capsule) were located in the stack of serial section images. Adhesion was defined as obvious continuity between the glomerular extracellular matrix and Bowman's capsule. Thus, by definition, subtle attachments of capillary loops to Bowman's capsule through bridging podocytes were not called “adhesions”, while segmentally sclerotic lesions included “adhesions”. A target grid with equal angular (π/8) and distance (10 µm) divisions (**Figure 3**) was superimposed on the glomerular profile containing the middle section passing through GTJ, while the grid center was at the Bowman's capsule center and its guidelines were parallel to the reference axis (**Figure 2**). The distance from GTJ to the grid center (*l_P_*) and the angle (*θ_P_*) made between the GTJ, grid center and the grid line perpendicular to the reference axis (origin axis) were recorded. Similarly, the target grid was superimposed on the glomerular profile containing the middle section through an adhesion, while the grid center was at the Bowman's capsule center and the guidelines were parallel to the reference axis (**Figure 2**). The distance from the adhesion center to the grid center (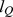) and the angle (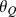) made between the adhesion, grid center and the origin axis were recorded. The distance between GTJ and the adhesion was calculated using the equation:
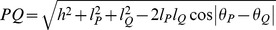
where 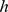 is the vertical distance between the two sections, calculated from the section thickness. Glomerular radius (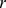) was determined on serial sections. It should be noted that known consistent section thickness is essential to obtain accurate results. In order to make 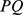 distances in different size glomeruli comparable, the values were expressed as 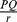. In order to prepare a graphical map of adhesions, a virtual sphere representing Bowman's capsule with tubular and vascular poles was divided into 6 zones of equal height, and hence of equal surface area, parallel to the equator. The equal surface area of each zone provides an equal probability for occurrence of a random adhesion to Bowman's capsule. The zones were numbered sequentially from GTJ (zone I) to the vascular pole (zone VI). 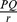 for each zone (0<zone I≤0.82; 0.82<zone II≤1.16; 1.16<zone III≤1.41; 1.41<zone IV≤1.63; 1.63<zone V≤1.83; and 1.83<zone VI≤2) was calculated and the number of adhesions per each zone was determined.

### Practical example

Based on the above-mentioned instructions and **Figure 1**, the distance from the adhesion to GTJ is calculated as follows:


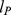
 (the distance from GTJ to the grid center): 10 (distance class)×10 µm (length of each class) = 100 µm


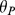
 (the angle between the GTJ, grid center and the origin axis): 5 (angle class)×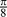 (angle of each class) = 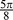



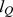
 (the distance from the adhesion center to the grid center): 9×10 µm = 90 µm (see above description for 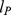)


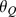
 (the angle between the adhesion, grid center and the origin axis): 9×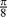 = 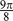 (see above description for 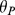)


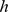
 (the vertical distance between the two profiles containing the GTJ and adhesion centers) = 3 (the number of sections apart)×5 µm (section thickness) = 15 µm

Plugging values of 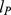, 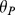, 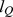, 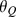, and 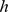 into the equation 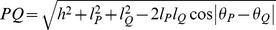:


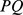
 (the distance between adhesion and GTJ) = 18 µm


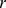
 (the radius of the glomerulus, estimated on serial sections): 120 µm


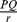
 = 0.15, therefore this adhesion falls into zone I, adjacent to GTJ.

### Equations and grid construction

**Figure 4:**
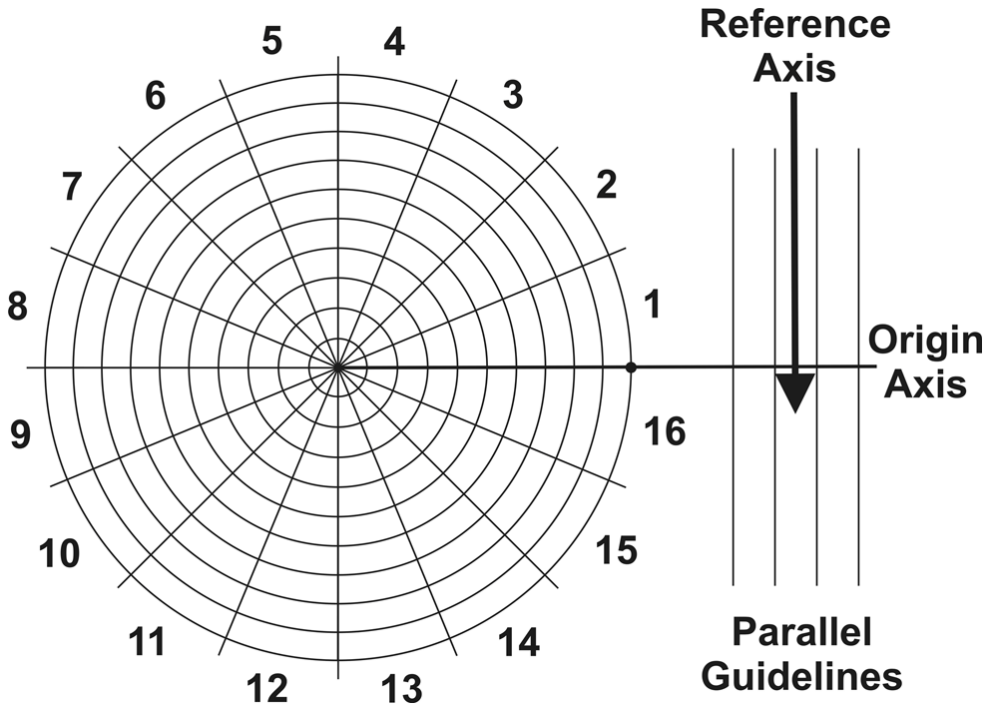
A sphere representing an ideal glomerulus cut by two parallel planes. The sphere with center 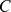 is intersected by two profiles (target grids and profiles of the Bowman's capsule) with centers 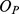 and 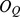. 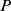 is a landmark on the surface (glomerulotubular junction or the vascular pole). 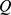 is an arbitrary point inside the spheres (the centroid of the segmental lesion). 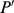 is the vertical projection of 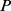 on the plane of 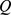.

Consider a sphere with center (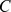) and radius (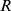) with a fixed point (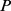) on its surface (**Figure 4**). 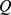 is an arbitrary point in the sphere (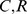). The distance 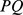 can be calculated using the information on two aligned parallel sections of known distance apart containing 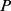 and 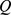:

If 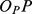 is vertically projected onto the profile containing 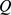 (dashed lines):
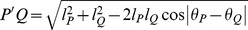
where 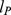 is the distance from the profile center containing 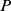 to the point 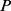; 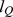 is the distance from the profile center containing 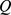 to the point 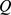; 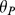 is the angle between an arbitrary fixed axis (i.e., reference axis) used to appropriately align serial sections, and the line connecting the center of the sphere profile containing 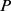 to the point 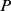; and 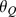 is the angle between the reference axis and the line connecting the center of the sphere profile containing 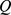 to the point 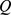.

And since
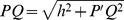
where 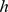 is the vertical distance between the two sphere profiles containing 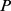 and 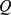 thus,
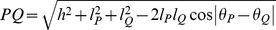
and the distance of 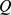 to the center of sphere (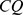)
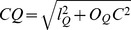



In order to obtain the information required to calculate 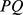 and 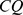, a target grid with 16 equal angular (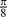 each) and 10 equal distance (10 µm each) divisions was developed (**Figure 3**). The grid is superimposed on the section containing 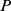 (GTJ in this example), while the grid center is at the Bowman's capsule center. The guidelines of the grid are aligned parallel to the section's reference axis. Values of 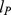 and 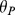 are recorded. The same procedure is repeated on the section containing 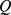 (adhesion in this example) to obtain 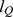 and 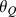.

### Validation of the geometrical model

The proposed model is based on the assumption of a spherical shape for Bowman's capsule. In order to test the validity of this assumption, the volume contained within the Bowman's capsule of 14 arbitrarily selected glomeruli were estimated using the Cavalieri principle [6] and separately using the sphere radius obtained from the number of sections passing through the entire Bowman's capsule. The values obtained from these two methods were compared using paired t-test and correlated using Pearson linear correlation. p-value<0.05 was considered statistically significant.
